# The Path of Science

**DOI:** 10.5812/atr.19125

**Published:** 2014-03-30

**Authors:** Esmaeil Fakharian

**Affiliations:** 1 Trauma Research Center, Kashan University of Medical Sciences, Kashan, IR Iran

**Keywords:** Trauma

Development of the science has paved a very slow and sometimes fluctuating way during the human life. Medicine with its complicated cultural, religious, and social aspects is not an exception to this rule. Most of our knowledge about the diseases and their impact on human is our understandings and explanations on the base of nowadays science. There is no specific and definite record about them by our ancestors before three thousand years ago and very vague as well as confusing data up to about two hundred years ago. On the base of the archeological findings, we suppose that disease process has a background history of about seventy-two million years. This estimation is based on the bony deformities and healed fractures found in fossils of a dinosaur called Gorgosaurus. Paleopathologic evaluation of the animal directed to the possibility of a cerebellar tumor which supposedly had resulted in its instability and repeated fallings and fractures. This process continues with the findings of many trapanized human skulls from different ages with evidence of healing (1); optimistically, it is supposed that they are therapeutic interventions. Recording was not possible in these cases. The story is more interesting when the events in the past three thousand years are reviewed. While recording of data was possible and they are present from different parts of the world, but we do not know how and why human being has used four elements, or in some cultures two elements, for the explanation of the events including diseases. It is even unknown how this concept was accepted and spread to different cultures and how it evolved and used for management of various diseases for more than two millennia. Limited number of the scholars, poor facilities for communication among them, and lack of thought flexibility as well as absence of a basic popular knowledge made the challenge toward a clear and fair concept very difficult if not impossible. The conflicts between scientists, their new ideas or different explanations about the natural processes, inquisition courts in renaissance and historical facts concerning trial and sentence of scientists in that period although disappointing and sadly events, are bright documents of such an atmosphere.

In this situation, many of the discoveries or findings and inventions were forgotten by the death of their owners, lost amongst their notes, or, by chance, repeated by the others and presented again. It is told that one of the earliest guidelines for experimental studies was recommended by Avicenna, the great Iranian philosopher and physician, in his Canon (2). In spite of this, the number of such studies, if happened at all, is scanty and rare data can be found in the literature in his era and then. A major cause and logical explanation for these problems may be the absence of communication through documents and records among scholars and societies. One of the brilliant points of the Iranian-Islamic civilization and scientific development in eighth to 14th centuries is the great number of books written or translated by the scientists and the very large number of libraries in many different cities all around the Islamic world including Iran. For any reason, which is not the scope of this article, one of the explanations of discontinuation of that bright period is deviation from recording of the experiments and experiences accompanied by a decline in scientific activities. This process went on in reverse direction in western countries after 14th century and the scientists followed the path of their eastern colleagues. The first academic journal published in Europe was Journal des Scavans in 1665 by Denis de Sallo (3). In 1731, Royal Society of Edinbrough published the first peer-reviewed journal called Medical Essays and Observations (4). Since then, the number of publishing journals has had an increasing pattern. With the worldwide technological advances in the past few decades, the opportunity for communication and presentation of the ideas has grown and this is one of the hallmarks and differences of the new world with the past. It has resulted in very rapid growth of knowledge and science in all aspects of human life and in all levels of the societies, even the poorly developed ones. Our journal as an academic journal with two years background of publication in the field of trauma has obtained the chance of indexing in “PubMed” as one of the most famous and widespread available resources of medical information and this is our honor to be accessible in different parts of the world. As a developing country with a very strong and well-known scientific background, we hope and try to help our country to approach its golden era. Everyone with the interests and apprehensions about trauma as one of the major threats of health in every part of the world is invited to contribute in this accepted and successful human design of growth and development.
